# 

**Published:** 1898-03

**Authors:** 


### NOTICES.

### American Dental Society of Europe.

The next meeting of the American Dental Society of Europe, which celebrates the 25th anniversary of the founding of the society, will take place in London during the first days of August of this year, and promises, so far, to be the most important and interesting meeting the society has ever held.

The place of meeting, and the exact date, will be decided upon in the near future and notice of same will be duly given.

It is particularly desirable that the meeting of this society in London should be one especially full and fruitful.

As the time for the meeting is now drawing near, it would be of much advantage, and also a special favor to me, if you could let me know very soon if you will contribute to this meeting either a paper, demonstration, or clinic, and which?

Hoping for an early reply in the affirmative, stating the subject as you wish it to appear on the programme,

I am yours fraternally,

L. A. O'BRIAN, *Secretary.*

DRESDEN, January, 1898.

### Illinois State Dental Society.

The thirty-fourth annual meeting of the Illinois State Dental Society will be held at Springfield, May 10–13, 1898. Dentists practicing in the State of Illinois, who are not members of the society, and dentists of other States are cordially invited to attend. Hotels and railroads will make the usual reduction. A large attendance is desired and a profitable meeting is anticipated.

A. H. PECK, *Secretary,*

CHICAGO, March 1, 1898. 92 State Street.

### Next Meeting of the American Medical Association, June 7th to 10th, 1898.

At a recent meeting of the Committee of Arrangements the following has been reported: The medical profession of the city have subscribed the amount of $6,000. The Committee on Meetings reported that the Gulf Railroad has published 20,000 copies of a pamphlet entitled "Colorado, About its Climate." The Committee on Hotels has gotten out an attractive leaflet giving the hotels and principal boarding-houses of the city. The Committee on Souvenir Book reported they have in preparation an exceedingly attractive souvenir volume, profusely illustrated, giving information on every imaginable subject of interest in the State, as well as a map of the State and city. It is confidently expected that the railroads will furnish an excursion "over the loop" and to Colorado Springs and Manitou, and that half rates will be charged for transportation and a time limit on all railroad tickets, of thirty days from the date of arrival.

### EDITORIAL.

### Tri-State Meeting.

The triennial meeting of the Tri-State Association will be held at Put-in-Bay, June 14th, 15th, and 16th, 1898. The time fixed for this meeting is probably satisfactory to all concerned, as to the place, the membership of the Ohio State Society selected this by a large majority, and the joint committee of three States have agreed to this selection.

These two questions being settled, it now behooves all interested to put forth every effort to make the meeting a success.

While Michigan and Indiana will doubtless do everything they can, Ohio should put forth every *possible* effort to make the occasion equal and superior, if possible, to the meeting at Detroit three years ago. The executive committee is putting forth every effort to make this a most inviting time. Every attraction available will be utilized.

The social feature will not be overlooked. The scientific and practical work is being amply provided, such as clinics, demonstrations, papers, addresses and discussions. The intention is to have a goodly number of prominent men of the profession from other States in attendance. There will doubtless be a liberal supply of instruments, appliances and materials, for exhibition and illustration; these things are always attractive.

The interval (three years) between these meetings is such that an unusual effort should be made by those interested, to make the occasion one of great interest and improvement. The accommodations will be of the first order. The method of reaching this historic spot will be given more in detail in the next number of the REGISTER; in the meantime, let everybody begin arranging their affairs with a view of being present at this great meeting.

### The Dental Register,

**A Monthly Magazine Devoted to the Interests of the**

### DENTAL PROFESSION.

*Terms Reduced to* $2.00 *Per Year. Single Copies,* 25 *Cents.*

**EDITED BY PROF. J. TAFT, D.D.S.**

AND

**Published by SAM'L A. CROCKER & CO., Ohio Dental and Surgical Depot.**

The volume commences in January and closes in December.

The Register being issued monthly is always fresh, therefore Indispensable to the practitioner who desires to keep posted up with the new inventions and improvements which are daily developed. Neither expense nor effort will be spared to give value and variety to its contents. Articles will be illustrated with well executed engravings whenever they will add to the interest or assist to explanation.

Its extensive circulation and frequency of issue make it one of the BEST DENTAL ADVERTISING MEDIUMS in the United States.

All subscriptions must begin with January or July.

Local or special notices, five lines or less, will be inserted for $3 each insertion; over five lines, 50 cts. per line.

All advertising bills payable in advance, or at furthest, after the issue of the first number containing the advertisement. All transient advertisements to be paid for in advance.

☞CONTRIBUTIONS SOLICITED.

Exclusively Editorial Communications should be addressed to the Editor.

The latest number of the Register may be ordered with or without other goods at any time, and all letters should be addres[illegible]

